# Quantitative Water Permeability Mapping of Blood-Brain-Barrier Dysfunction in Aging

**DOI:** 10.3389/fnagi.2022.867452

**Published:** 2022-04-08

**Authors:** Jeremy N. Ford, Qihao Zhang, Elizabeth M. Sweeney, Alexander E. Merkler, Mony J. de Leon, Ajay Gupta, Thanh D. Nguyen, Jana Ivanidze

**Affiliations:** ^1^Department of Radiology, Massachusetts General Hospital, Boston, MA, United States; ^2^Department of Radiology, Weill Cornell Medicine, New York, NY, United States; ^3^Department of Biostatistics, University of Pennsylvania, Philadelphia, PA, United States; ^4^Department of Neurology, Weill Cornell Medicine, New York, NY, United States

**Keywords:** arterial spin labeling, blood-brain barrier, aging, glymphatic, cerebral small vessel disease

## Abstract

Blood-brain-barrier (BBB) dysfunction is a hallmark of aging and aging-related disorders, including cerebral small vessel disease and Alzheimer’s disease. An emerging biomarker of BBB dysfunction is BBB water exchange rate (k_W_) as measured by diffusion-weighted arterial spin labeling (DW-ASL) MRI. We developed an improved DW-ASL sequence for Quantitative Permeability Mapping and evaluated whole brain and region-specific k_W_ in a cohort of 30 adults without dementia across the age spectrum. In this cross-sectional study, we found higher k_W_ values in the cerebral cortex (mean = 81.51 min^–1^, *SD* = 15.54) compared to cerebral white matter (mean = 75.19 min^–1^, *SD* = 13.85) (*p* < 0.0001). We found a similar relationship for cerebral blood flow (CBF), concordant with previously published studies. Multiple linear regression analysis with k_W_ as an outcome showed that age was statistically significant in the cerebral cortex (*p* = 0.013), cerebral white matter (*p* = 0.033), hippocampi (*p* = 0.043), orbitofrontal cortices (*p* = 0.042), and precunei cortices (*p* = 0.009), after adjusting for sex and number of vascular risk factors. With CBF as an outcome, age was statistically significant only in the cerebral cortex (*p* = 0.026) and precunei cortices (*p* = 0.020). We further found moderate negative correlations between white matter hyperintensity (WMH) k_W_ and WMH volume (*r* = −0.51, *p* = 0.02), and normal-appearing white matter (NAWM) and WMH volume (*r* = −0.44, *p* = 0.05). This work illuminates the relationship between BBB water exchange and aging and may serve as the basis for BBB-targeted therapies for aging-related brain disorders.

## Introduction

The blood-brain barrier (BBB) is comprised of endothelial cells connected by tight junctions, pericytes, and astrocytic end-feet, and regulates homeostasis of fluid and solutes at the blood-central nervous system (CNS) interface (Ballabh et al., 2004). Converging evidence suggests that BBB dysfunction plays a central role in the aging brain (Weiss et al., 2009; Banks et al., 2021).

One emerging sensitive probe of BBB water permeability is through imaging-based measurement of water exchange across the BBB (Dickie et al., 2020). Vascular compromise in AD is reflected in impaired transport of water across the blood-CSF and CSF-brain barriers. Mechanistically, the system is impacted by age as are the cellular vascular membrane properties. Further, Aquaporin-4 (AQP4) water channels localizing to perivascular astrocytic endfeet (also known as AQP4 polarization) form a central pathway for the glymphatic system, enabling water transport across the BBB (Haj-Yasein et al., 2011; Iliff et al., 2012; Hladky and Barrand, 2014; MacAulay, 2021). Decreased AQP4 polarization is associated with aging and with β-amyloid (Aβ) deposits in animal models (Yang et al., 2011; Kress et al., 2014; Ishida et al., 2020) and humans (Zeppenfeld et al., 2017). This feature cannot be adequately characterized with dynamic contrast-enhanced (DCE) MRI due to the molecular properties of a Gadolinium-based contrast agent (GBCA), given that GBCA are several orders of magnitude larger than water and unlike water cannot enter the brain parenchyma via transcellular route. BBB permeability to GBCA mapped with DCE MRI, expressed as K_TRANS_, has been shown to be mildly increased at baseline in the hippocampus of young healthy subjects (Ivanidze et al., 2019). Moreover, hippocampal K_TRANS_ elevation has been shown to be associated with normal aging (Montagne et al., 2015), and more recently has been implicated in age-related cognitive dysfunction (Bowman et al., 2018; Nation et al., 2019) and Alzheimer’s disease (AD) (Halliday et al., 2016; Van De Haar et al., 2016; Montagne et al., 2021). However, K_TRANS_ primarily measures the paracellular leakage of the relatively large GBCA molecules through the endothelial tight junctions (Laurent et al., 2006) and cannot capture the transcellular transport of the much smaller water molecules through AQP4 channels on astrocyte end-feet (Solenov et al., 2004; Papadopoulos and Verkman, 2005; Kress et al., 2014; Ma et al., 2017), nor via other co-transport mechanisms (Steffensen et al., 2018). Furthermore, electrophysiological experiments demonstrated that BBB permeability to macromolecules such as albumin is not directly correlated to BBB permeability for small ions such as potassium (Kang et al., 2013).

An emerging technique to image BBB water exchange rate (k_W_) is diffusion-weighted arterial spin labeling (DW-ASL), an approach that obviates the need for GBCA injection. DW-ASL techniques have been employed to evaluate changes in k_W_ in obstructive sleep apnea (Palomares et al., 2015), cerebral small vessel disease (CSVD) (Shao et al., 2019), and ischemic infarction (Tiwari et al., 2017). While promising, the previously developed gradient spin echo (GRASE) based DW-ASL approaches for *in vivo* imaging suffer from low spatial resolution, limited brain coverage, and off-resonance artifacts particularly at higher static field strengths. Furthermore, it is unclear whether water exchange increases or decreases with age; given that water transport into the brain parenchyma may occur via either transcellular water channel regulated by aquaporin-4 (AQP4) (the expression of which decreases with age) or paracellular BBB leakage (which increases with age), or a combination of both.

We have recently developed an improved DW-ASL sequence based on the more robust stacks-of spirals 3D fast spin echo (FSE) data acquisition and adiabatic diffusion preparation, termed Quantitative Permeability Mapping (QPM) (Zhang et al., 2020) that mitigates existing technical challenges. The purpose of this study was to demonstrate the relationship between age and region-specific BBB water exchange, as measured by k_W_ using QPM sequence, in normal volunteers across the age spectrum.

## Materials and Methods

### Ethics Statement, Subject Recruitment, Selection, and Consent

Following institutional review board approval and written informed consent, 36 volunteers aged 25 years and above who had previously expressed their interest in participating in brain imaging research were contacted. Thirty volunteers agreed to participate in the study. Based on subject interview and electronic medical record review, none of the volunteers satisfied exclusion criteria, which included the following: medical history of neurodegenerative disorder, chronic territorial infarction, illicit substance abuse disorder, neuropsychiatric disorder, cerebrovascular accident, or traumatic brain injury. To obtain an even distribution of ages, we aimed to recruit approximately 10 subjects per age group 25–44, 45–64, and 65+.

### Vascular Risk Factor and Cognitive Assessment

Using subject interview and electronic medical record review, subjects were evaluated for four vascular risk factors: hypertension, hyperlipidemia, type 2 diabetes mellitus, and tobacco use. These risk factors were selected given that they are among the most common contributors to microvascular disease (Khan et al., 2007). Subjects were evaluated on the presence or absence of vascular risk factors (see section “Statistical Analysis” below).

All subjects were evaluated in-person with the Montreal Cognitive Assessment (MoCA) version 8.1 (English). MoCA is a screening test designed to detect subjects with mild cognitive impairment (MCI) and dementia. The performing physician (JF) was certified by MoCA Test Inc., to administer the MoCA examination in the standardized, validated format, including the 0–15-point Memory Index Scale (MIS), allowing to identify subjects at risk for dementia (Julayanont et al., 2012). For both the MoCA and MIS, higher scores indicate more correct items on the examination (Nasreddine et al., 2005).

### Quantitative Permeability Mapping Sequence and MRI Scanning Protocol

All scans were performed on a single GE Discovery 3.0T 750 MRI system using a product 32-channel head coil for signal reception. To mitigate the heterogeneity of caffeine effects on perfusion and water permeability (Wengler et al., 2019), subjects abstained from caffeine for at least 3 h prior to image acquisition, a pre-scan protocol which has been reported previously (Shao et al., 2019).

Our QPM sequence (Zhang et al., 2020) is based on a signal-to-noise ratio-efficient 3D stack-of-spirals FSE acquisition developed previously for pseudo-continuous ASL (pCASL) imaging (Dai et al., 2008) and can achieve whole brain coverage with a 1.9 × 1.9 × 4 mm^3^ resolution. Spiral FSE readout was used to enhance robustness against off-resonance artifacts at 3T field strength (Alsop et al., 2015). This was combined with a BIR-4 adiabatic pre-pulse which provides more robust diffusion preparation than the more commonly used composite hard pulse design at 3T (Nguyen et al., 2016).

QPM data acquired at multiple post-labeling delays (PLD) and *b*-values were used to calculate k_W_, cerebral blood flow (CBF), and arterial transit time (ATT) by fitting a two-compartment signal model as follows:


(1) $$I=△M_{b}\left(t\right)+\left(1-sign\left(b\right)\right)△M_{c}\left(t\right)$$

(2) $$△M_{b}\left(t\right)=-\frac{2CBF\varepsilon M_{0}\beta }{\lambda }[\frac{e^{-\left(R_{1a}-R_{1b}\right)ATT}}{R_{1b}}\left(e^{-R_{1b}\left(t-\delta \right)}-e^{-R_{1b}t}\right)-\frac{e^{-\left(R_{1a}-\alpha \right)ATT}}{\alpha }\left(e^{-\alpha \left(t-\delta \right)}-e^{-\alpha t}\right)]$$

(3) $$△M_{c}\left(t\right)=-\frac{2CBF\varepsilon M_{0}e^{-\left(R_{1a}-R_{1b}\right)ATT}}{\lambda \alpha }\left(e^{-\alpha \left(t-\delta \right)}-e^{-\alpha t}\right)$$

Here, *I* is the acquired QPM image, △*M*_*b*_(*t*) and △*M*_*c*_(*t*) are signals in tissue and capillary compartments, respectively, sign(b) is 1 if diffusion preparation is applied and 0 otherwise, ε = 0.6 is the product of the initial labeling efficiency (0.8) and the loss of labeling due to the background suppression pulses (0.75) (Mutsaerts et al., 2014), *M_0_* is reference proton density image, λ = 0.9 mL/g is the blood-brain partition coefficient (Alsop et al., 2015), *R*_*1a*_ and *R*_*1b*_ are the longitudinal relaxation rates of blood and brain tissue, respectively [*R*_1*a*_ = 0.6*s*^−1^ (Lu et al., 2004), *R*_*1b*_ is calculated using a FAST-T1 mapping sequence (Nguyen et al., 2017)], δ = 1.5 s is the labeling time, and *t* = δPLD, α = *k*_*W*_*R*_1*a*_ and $\beta =\frac{k_{W}}{k_{W}R_{1a}-R_{1b}}$. Given QPM images *I_1_* to *I_N_* acquired with different PLDs and *b*-values, k_W_, CBF, and ATT maps can be fit from Eqs 1–3 by minimizing the following cost function with L2 regularization:


(4) $$k_{W}^{*},CBF^{*},ATT^{*}=\underset{k_{W},CBF,ATT\;}{\arg\min}\sum_{i=1}^{N}||\gamma_{i}\left(I_{i}-f\left(k_{W},CBF,ATT\right)\right)|{|}_{2}^{2}\mu ||k_{W}|{|}_{2}^{2}\mu ||CBF|{|}_{2}^{2}\mu ||ATT|{|}_{2}^{2}$$

k_W_, CBF and ATT maps were generated from a multi-PLD multi-b QPM scan acquired with *b* = 20 s/mm^2^ at PLD = 1,000, 1,500, 1,800, 2,000, and 2,500 ms [number of excitations (NEX) = 3] and *b* = 0, 10, 20, 50, 100 s/mm^2^ at PLD = 1,200 (NEX = 1) ms in 27 min. The optimal regularization parameter μ was chosen based on the L-curve method (Hansen, 1992), and the noise weighting term was set as $\gamma_{i}=\sqrt{\text{NEX}}$. Equation 4 was solved using a custom iterative gradient descent algorithm with the maximum number of iterations of 100 and the tolerance of relative change in the solution set to 0.01. For initialization, k_W_ map was set to 0, CBF map was calculated from QPM scan acquired at PLD = 1,500 ms (Mutsaerts et al., 2014), and ATT map was calculated as the signal-weighted PLDs as previously described (Dai et al., 2012).

Additionally, a 3D T1-weighted BRAVO sequence was obtained for anatomic definition, and a 3D T2-weighted FLAIR (T2-FLAIR) sequence was acquired in 20 of the 30 subjects to identify and quantify white matter hyperintensity (WMH). T2-FLAIR imaging was reserved for subjects 45+ years old given than WMH would be expected to be rare in healthy subjects under 45 years old. To minimize partial volume effects from small areas of WMH, a threshold of WMH volume of 100?mm^3^ (0.1 cc, approximately 5 mm in linear dimension) was set as inclusion criteria for the analysis based on the recommendation that lesion size should be at least 5 times larger than the voxel size for lesion geometry to be captured reliably and also to minimize the effect of imperfect coregistration between scans (Firbank et al., 1999).

### Region of Interest Segmentation and Analysis

The following regions selected for analysis and bilateral regions were analyzed as a single region of interest (ROI): cerebral cortex, cerebral white matter, hippocampi, precunei cortices, and orbitofrontal cortices. The regions selected show differential vulnerability in aging and AD, with the hippocampus and precuneus typically demonstrating greater metabolic and perfusion effects (Cavedo et al., 2014; Riederer et al., 2018). FreeSurfer (Dale et al., 1999) (Charlestown, Massachusetts; Massachusetts General Hospital) was used to obtain brain ROI segmentation from the T1-weighted BRAVO anatomical image. The k_W_, CBF, and ATT values for each ROI was obtained from the corresponding co-registered parametric maps in ITK-SNAP using FreeSurfer-generated brain labels.

### White Matter Hyperintensity Segmentation

In the 20 subjects with T2-FLAIR image, WMH within the cerebral white matter was manually segmented in ITK-SNAP (Yushkevich et al., 2006)^1^ by a neuroradiology fellow (JF). To obtain WMH-specific average k_W_ and CBF values, the WMH masks were linearly coregistered to k_W_ and CBF maps using FSL FLIRT command (Jenkinson et al., 2002) with rigid-body motion (translation and rotation) and trilinear interpolation followed by thresholding at 0.5 probability level.

### Statistical Analysis

All statistical analyses were performed in GraphPad Prism 9.1.1 (GraphPad Software, San Diego, CA, United States). The non-parametric Wilcoxon signed-rank test was used to compare k_W_ values measured in the cerebral cortex and cerebral white matter, and the non-parametric Kruskal-Wallis test with a Dunn’s test for multiple comparisons was used to compare k_W_ values between hippocampi, orbitofrontal cortices, and precunei cortices. To determine the association between k_W_ and age, a multiple linear regression model with k_W_ measured in the cerebral cortex, cerebral white matter, hippocampi, orbitofrontal cortices, and precunei cortices as an outcome and age, sex, and the total number of vascular risk factors (minimum 0, maximum 4) as predictors. The same analysis was then repeated for CBF and ATT. The non-parametric Mann-Whitney *U*-tests were used to compare k_W_ values in subjects with and without vascular risk factors. Pearson correlations were performed to evaluate the relationship between WMH k_W_ and WMH volume, and between normal-appearing white matter (NAWM) k_W_ and WMH volume, with corresponding analyses for CBF. To test the hypothesis that k_W_ is significantly different in WMH vs. NAWM in the same subject, a Wilcoxon signed-rank test was used for the 20 subjects with WMH volume over 100 mm^3^ (0.1 cc). *P*-values < 0.05 were considered as statistically significant.

## Results

### Demographic Characteristic of Study Participants

Age and sex distribution, cognitive scores, and vascular risk factors of study participants are outlined in Table 1.

**TABLE 1:** Clinical and demographic characteristics of the study population.

Age range (Years)	Number of subjects	Number (%) female	Mean MoCA (SD)	Mean MIS (SD)	Number (%) with 1+ vascular risk factor
25–44	9	2 (22.2%)	29.3 (1.1)	14.1 (1.5)	0 (0.0%)
45–64	8	3 (35.7%)	27.0 (1.6)	10.5 (3.2)	3 (37.5%)
65+	13	6 (46.2%)	26.7 (3.0)	11.3 (4.4)	10 (76.9%)
All	30	11 (36.7%)	27.6 (2.5)	11.9 (3.6)	13 (43.3%)

*All subjects had at least 4 years of education after high school. MoCA scores (0–30; 27+ = normal, 17–25 = mild cognitive impairment, < 17 = dementia) tended to decrease with age. Four subjects, all 65 years or older, had scores compatible with mild cognitive impairment, the youngest being 51 years old. No subjects had a MoCA score compatible with dementia. Risk factors queried with hypertension, hyperlipidemia, diabetes, and smoking (current or former). Older subjects tended to accumulate more vascular risk factors.*

### Age-Related Regional Differences in k_W_ and Cerebral Blood Flow

**FIGURE 1:**
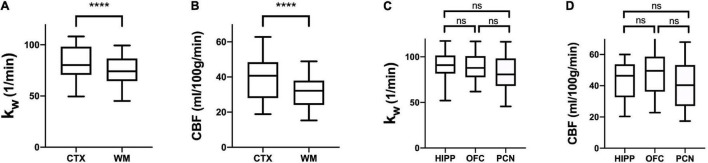
Regional k_W_ and CBF. Tukey box plots of k_W_ **(A,C)** and CBF **(B,D)** values obtained in the cerebral cortex (CTX), cerebral white matter (CWM), hippocampi (HIP), orbitofrontal cortices (OFC), and precunei cortices (PCN) (*n* = 30). CTX exhibited higher k_W_ and CBF than CWM (**** indicates *p* < 0.0001, non-parametric Wilcoxon signed-rank test), whereas there were no significant differences between HIP, OFC, and PCN (Kruskal-Wallis test with a Dunn’s test for multiple comparisons). ns, not significant.

Regional Analysis: k_W_ values were consistently higher in cerebral cortex (mean = 81.51 min^–1^, *SD* = 15.54) compared to cerebral white matter (mean = 75.19 min^–1^, *SD* = 13.85) in all subjects (*p* < 0.0001), indicating greater BBB water exchange. CBF values were also higher in the cortex (mean = 39.60 mL/100 g/min, *SD* = 12.44) relative to cerebral white matter (mean = 32.47 mL/100 g/min, *SD* = 9.18) (*p* < 0.0001). In sub-lobar cortical regions, there were no significant differences between the hippocampi, orbitofrontal cortices, and precunei cortices with respect to either k_W_ or CBF (Figure 1).

#### Age Effects

**FIGURE 2:**
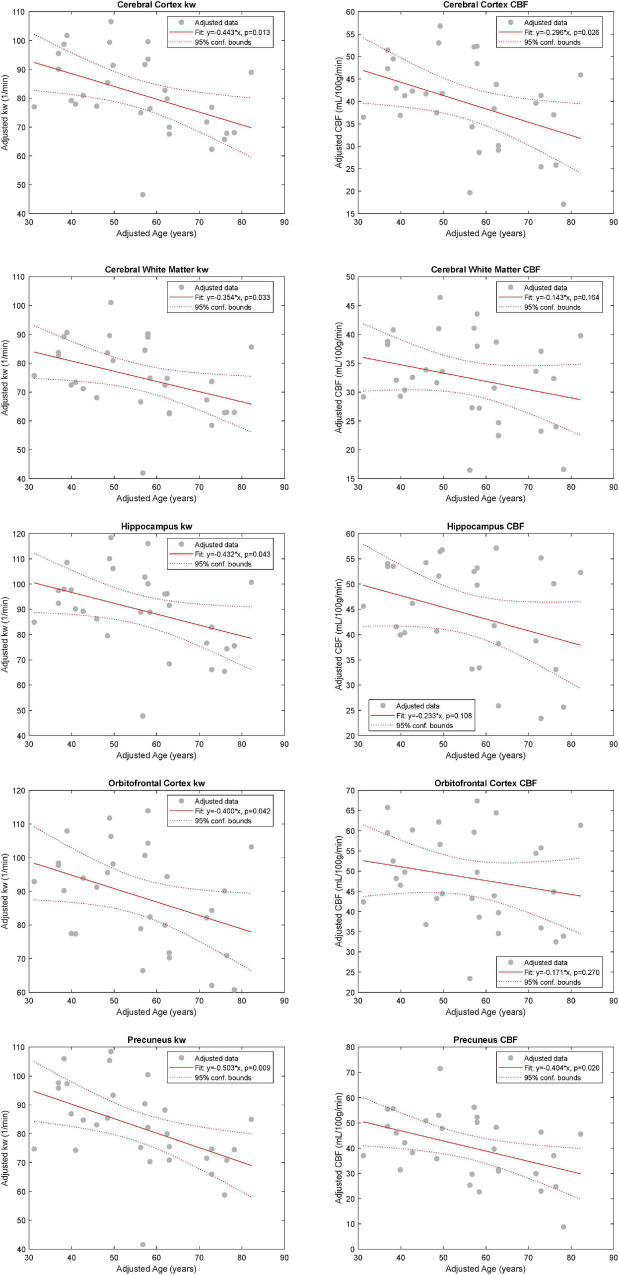
Relationship between k_W_ and CBF with Age. Added variable plots obtained by multiple linear regression analysis showing the relationship between k_W_ and CBF with age (*n* = 30), adjusting for sex and the number of vascular risk factors. There was a statistically significant association between age and k_W_ in all five evaluated regions: cerebral cortex (CTX), cerebral white matter (CWM), orbitofrontal cortices (OFC), and precunei cortices (PCN). With respect to CBF, age was found to be statistically significant only in CTX and PCN.

**FIGURE 3:**
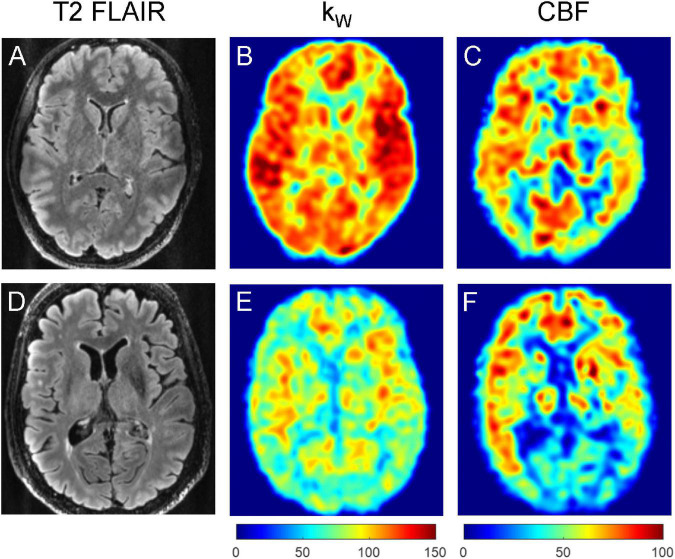
Representative T2-FLAIR images **(A,D)**, k_W_ maps **(B,E)**, and CBF **(C,F)** maps obtained from a 32-year-old subject with no vascular risk factors **(A–C)** and a 77-year-old subject with one vascular risk factor (former smoker, 40 pack-year) **(D–F)**. The younger subject demonstrated mean cortical k_W_ of 108.1 min^–1^, mean white matter k_W_ of 97.4 min^–1^, mean cortical CBF of 60.1 mL/100 g/min, and mean white matter CBF of 48.6 mL/100 g/min. The older subject demonstrated lower mean cortical k_W_ of 74.8 min^–1^, mean white matter k_W_ of 70.6 min^–1^, mean cortical CBF of 44.7 mL/100 g/min, and mean white matter CBF of 37.9 mL/100 g/min.

In the multiple linear regression model with k_W_ as an outcome, age was found to be statistically significant in the cerebral cortex (β = −4.43 min^–1^/decade, *p* = 0.013), cerebral white matter (β = −3.54 min^–1^/decade, *p* = 0.033), hippocampi (β = −4.32 min^–1^/decade, *p* = 0.043), orbitofrontal cortices (β = −4.00 min^–1^/decade, *p* = 0.042), and precunei cortices (β = −5.03 min^–1^/decade, *p* = 0.009), after adjusting for sex and number of vascular risk factors. With CBF as an outcome, age was found to be statistically significant only in the cerebral cortex (β = −2.96 mL/100 g/min/decade, *p* = 0.026) and precunei cortices (β = −4.04 mL/100 g/min/decade, *p* = 0.020) (Figure 2). Age was also statistically significant in the model with ATT as an outcome in the cerebral cortex (*p* = 0.002), cerebral white matter (*p* = 0.008), orbitofrontal cortices (*p* = 0.021), and precunei cortices (*p* = 0.001), but not in the hippocampi (*p* = 0.113). Univariate linear regression analyses for k_W_ and CBF vs. age are shown in Supplementary Figure 1. Representative k_W_ maps, CBF maps, and T2-FLAIR images from two subjects are shown in Figure 3.

### Cognition Assessments

Results from the cognitive assessments revealed that no subject had a MoCA score < 17, a cutoff that has been previously validated as the threshold between mild cognitive impairment (MCI) and dementia (Trzepacz et al., 2015). Four subjects had a MoCA score compatible with MCI (score 17–25).

### Effects of Vascular Risk Factors and White Matter Hyperintensities

**FIGURE 4:**
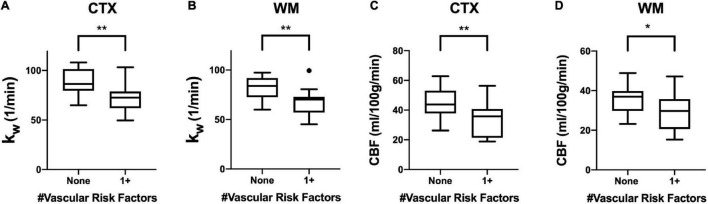
Regional k_W_ and CBF in the Context of Vascular Risk Factors. Tukey box plots showing **(A,B)** k_W_ values and **(C,D)** CBF values were significantly higher in both the cerebral cortex (CTX) and cerebral white matter (CWM) in subjects without vascular risk factors (smoking, diabetes mellitus, or hypertension) (*n* = 17) than those with at least one vascular risk factor (*n* = 13). (** indicates *p* < 0.01, * indicates *p* < 0.05, non-parametric Mann-Whitney *U*-test).

In our cohort, 13 of 30 subjects had at least one vascular risk factor. Five subjects had two vascular risk factors, and three subjects had three vascular risk factors. Values of k_W_ were lower among individuals with at least one vascular risk factor compared with those without in both the cerebral cortex (*p* = 0.002) and cerebral white matter (*p* = 0.007). CBF values were also significantly lower in the cerebral cortex (*p* = 0.006) and cerebral white matter (*p* = 0.025) (Figure 4).

### White Matter Hyperintensities, k_W_, and Cerebral Blood Flow

**FIGURE 5:**
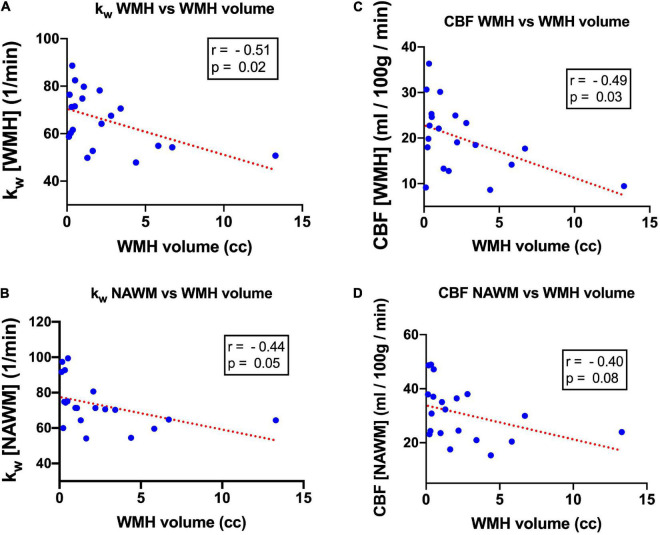
Scatterplot and linear regression line characterizing the relationship between k_W_ and WMH volume in **(A)** WMH and **(B)** NAWM, as well as the relationship between CBF and WMH volume in **(C)** WMH and **(D)** NAWM.

**FIGURE 6:**
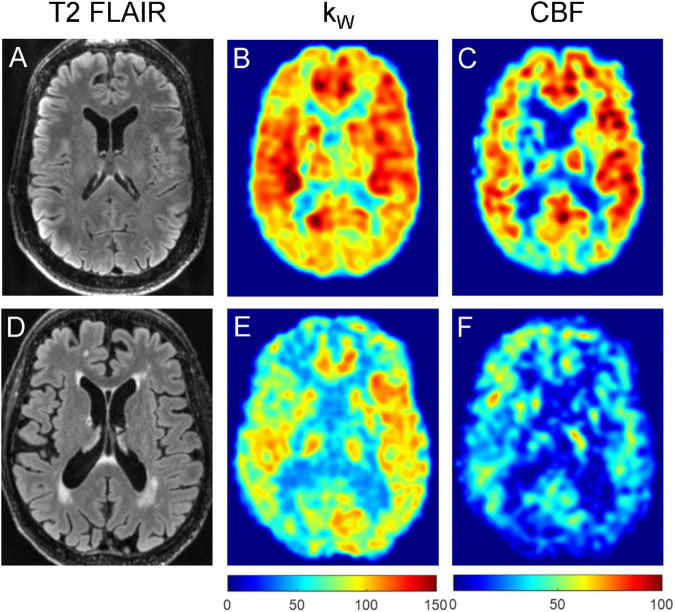
Representative T2-FLAIR images **(A,D)**, k_W_ maps **(B,E)** and CBF maps **(C,F)** obtained from a 76-year-old subject with no vascular risk factors and no significant WMH **(A–C)**, and a 74-year-old subject with two vascular risk factors (HTN, HLD) and moderate burden of WMH **(D–F)**. The subject without appreciable WMH demonstrated mean cortical k_W_ of 98.4 min^–1^, mean white matter k_W_ of 93.8 min^–1^, mean cortical CBF of 56.6 mL/100 g/min, and mean white matter CBF of 47.6 mL/100 g/min. The subject with moderate WMH burden (total WMH volume of 5,456 mm^3^) demonstrated lower mean cortical k_W_ of 72.6 min^–1^, mean white matter k_W_ of 64.0 min^–1^, mean cortical CBF of 28.1 mL/100 g/min, and mean white matter CBF of 23.6 mL/100 g/min.

For the 20 subjects who underwent T2-FLAIR imaging, 100% (20/20) of subjects had WMH volume above the 100 mm^3^ threshold (mean: 2,414 mm^3^, range: 127–13,293 mm^3^). There was a moderate negative correlation between WMH k_W_ and WMH volume (*r* = −0.51, *p* = 0.02) (Figure 5A). k_W_ within NAWM also demonstrated moderate negative correlation with WMH volume (*r* = −0.44, *p* = 0.05) (Figure 5B). There was a moderate negative correlation between WMH CBF and WMH volume (*r* = −0.49, *p* = 0.03) (Figure 5C); correlation between NAWM CBF and WMH volume did not reach statistical significance (Figure 5D). Representative images illustrating the relationship of k_W_ and CBF with WMH volume are shown in Figure 6.

## Discussion

In this study, we applied QPM, an improved DW-ASL sequence, to directly probe the relationship between aging and BBB water exchange in healthy adult volunteers. We found that BBB water exchange is lower among older adults, and in our pilot study sample of 30 subjects, age was significantly associated with k_W_ in all five selected brain regions. All evaluated regions demonstrated a negative correlation between age and k_W_, holding sex and number of vascular risk factors constant.

One ostensible mechanism for the negative association between age and k_W_ is via aquaporin-4AQP4. AQP4 is a water-selective cell membrane channel concentrated at perivascular astrocytic end-feet and is the prime mediator of diffusive and advective water transport in the brain (Simard et al., 2003; Nagelhus and Ottersen, 2013; Thomas, 2019). AQP4 serves an important function in the brain’s glymphatic system (Iliff and Simon, 2019), a mechanism for the elimination of soluble material from the CNS (Jessen et al., 2015). Accumulating evidence illustrates the role of alterations in AQP4 localization and expression in aging and aging-related disease; specifically, the loss of AQP4 polarization at the astrocytic end-feet (Abbott et al., 2006; Yang et al., 2011; Kress et al., 2014; Zeppenfeld et al., 2017). One possible mechanism highlighting the role of AQP4 in the aging brain begins with chronic perfusion stress and hypoxia (Bell et al., 2010; Sengillo et al., 2013), leading to loss of endothelial pericytes and astrocytic AQP4 polarization (Gundersen et al., 2014; Duncombe et al., 2017). The reduced localization of AQP4 at astrocytic end-feet could impair glymphatic function, with resultant misaggregation of proteins that drive aging-related CNS diseases. Our findings are concordant with emerging evidence that loss of AQP4 localization at astrocytic end-feet is a central feature of the aging BBB (Abbott et al., 2006; Yang et al., 2011; Kress et al., 2014; Zeppenfeld et al., 2017) and suggests that the effect of AQP4 localization on BBB water exchange predominates over BBB leakage in healthy adults.

Using GBCA DCE-MRI, multiple groups have observed increased BBB leakage with both normal aging (Wang et al., 2006; Xu et al., 2017; Verheggen et al., 2020) and aging-related neurodegenerative disorders (Sengillo et al., 2013; Montagne et al., 2015; Halliday et al., 2016; Van De Haar et al., 2016; Sweeney et al., 2018; Nation et al., 2019; Moon et al., 2021). Using a modified DCE-based approach with delayed acquisition, voxel-wise analysis and linear fitting to the late component of the concentration curve, Veksler et al. (2020) demonstrated the ability of DCE-MRI to visualize slow signal change following GBCA injection in traumatic brain injury and a variety of other pathologies. While k_W_ derived from GBCA-free DW-ASL has been described as an alternative to DCE-MRI to query BBB leakage (Shao et al., 2019), recent findings from a comparison study suggest that these two MRI techniques measure different aspects of BBB integrity (Shao et al., 2020). In this study, k_W_ was found to decrease with age, which shows an opposite trend compared to DCE-derived K_TRANS_. The mechanisms of BBB exchange of GBCA and water are likely to be independent given that AQP4 is not available to GBCAs as a pathway into the CNS. Animal models of ischemic cerebral infarction and mannitol-induced BBB opening a showed a direct relationship between DCE-MRI BBB leakage (expressed as K_TRANS_) and k_W_ (Tiwari et al., 2015, 2017). However, given the significant difference in body size and hemodynamics between rodents and humans, these results may not be directly translatable to the clinical setting. Furthermore, these scenarios represent acute BBB damage rather than chronic, adaptive changes in water exchange that may occur over time. This could partially explain the finding of Shao et al. (2020), given that only three brain regions demonstrated significant correlation between k_W_ and K_TRANS_ in an elderly cohort, the authors positing diverging BBB mechanisms mediating k_W_ and K_TRANS_. The relationship between and k_W_ and K_TRANS_ in normal aging as well as in neurodegenerative diseases in humans remains to be explored.

The physiologic basis of decreased k_W_ and decreased perivascular AQP4 localization with aging is not fully understood. In animal models of water intoxication and cerebral ischemic infarction, AQP4-deficient mice were found to have diminished resultant cerebral edema relative to wild-type mice, which led to improved survival and neurological outcomes (Manley et al., 2000). This role of AQP4 in mediating cerebral edema has been further validated by other groups in both animal models and humans (Taniguchi et al., 2000; Kleffner et al., 2008; Nakano et al., 2018). BBB disruption, either from acute (e.g., infarction, hemorrhage) or chronic, age-associated endothelial injury leads to a loss of tight-junctions and other components of the vascular BBB (Mooradian et al., 2003; Rosenberg and Yang, 2007; Kazmierski et al., 2012; Elahy et al., 2015; Keep et al., 2018; Stamatovic et al., 2019). In both the acute and chronic setting, this BBB leakage could lead to increased hydrostatic pressure in the perivascular space, rendering the brain vulnerable to cerebral edema. However, in the chronic setting, protective changes at the perivascular astrocytic end-feet may manifest through decreased perivascular AQP4 localization, leading to the observation of decreased k_W_ in our study. This adaptive response, however, may come with the cost of diminished glymphatic clearance, giving rise to protein misagreggation that promotes aging-related neurodegeneration (Iliff et al., 2012; Braun and Iliff, 2020; Nedergaard and Goldman, 2020). Conjecturally, it is conceivable that the enlarged perivascular spaces associated with dementia (Ding et al., 2017; Paradise et al., 2021) are a result of decreased BBB water exchange, consequently expanding the perivascular water reservoir.

Several previously published studies appear discordant with our findings; for example, some groups have demonstrated age-related *increases* in brain AQP4 expression with age (Gupta and Kanungo, 2013; Owasil et al., 2020). However, these studies do not specify whether measured AQP4 is localized to perivascular astrocytic end-feet, the site critical for BBB water exchange. In fact, Zeppenfeld et al. (2017) also found an increase in *global* AQP4 immunoreactivity in the frontal cortex with increasing amyloid burden, but observed decreasing *perivascular* AQP4 immunoreactivity with both amyloid burden and Braak stage. It is conceivable that increased non-perivascular AQP4 within the parenchyma represents an additional adaptive response to preserve a vestige of glymphatic flow.

While AQP4 plays a central role in water transport across the BBB, other important mechanisms have to be considered that may contribute to age-related changes in water permeability shown in our study. Co-transporter proteins have been shown to transport water along with ions such as potassium, sodium, and chloride, independently of an osmotic gradient. While such co-transporters, for example NKCC1, have been localized primarily in the choroid plexus, the exact regional distribution and the degree to which co-transporters contribute to water transport across the BBB, remains to be elucidated (Steffensen et al., 2018). Co-transporters have been demonstrated on the surface of endothelial cells and may thus contribute to water transport across the BBB (Hladky and Barrand, 2016).

It should be noted that recent studies in animal models of aging an AD suggest an *increase* in BBB water using alternative ASL-based techniques (Dickie et al., 2021; Ohene et al., 2021). However, multiple groups have pointed to significant interspecies differences in the content, structure, and function of the BBB (Syvänen et al., 2009; Warren et al., 2009; Deo et al., 2013; Hoshi et al., 2013), with greater concordance between humans and non-human primates. Moreover, owing largely to the two to fourfold relative decrease in CBF in rodents, the water extraction fraction is lower in rodents (Takagi et al., 1987) relative to humans (Paulson et al., 1977; Herscovitch et al., 1987) and monkeys (Eichling et al., 1974). These differences may have implications for adaptations that occur at the BBB with aging. Nonetheless, more work is needed to explain these divergent results between rodents and humans.

In addition to age-associated decreases in k_W_, we also found that k_W_ tends to decrease with volume of WMH burden, a relationship also observed with CBF in our cohort. We also noted that within WMH, k_W_ and CBF tended to be lower compared to NAWM, suggesting that BBB dysfunction and reduced perfusion may play a key role in the development of WMH. Zhang et al. (2019) using DCE-MRI demonstrated that BBB leakage volume within WML tended to increase with overall WML volume. The same authors also previously identified general BBB leakage in cerebral gray matter, NAWM, and WML that increased with in those with CSVD (Zhang et al., 2017). This association between BBB breakdown and CSVD is further validated in animal models (Schreiber et al., 2013; Kaiser et al., 2014), with one study showing that reversal of BBB dysfunction decreases white matter vulnerability to injury (Rajani et al., 2018).

In addition to genetic factors, vascular risk factors are primary drivers of CSVD (Staals et al., 2014; Power et al., 2015; Gyanwali et al., 2019), concordant with our observation that subjects with at least one vascular risk factor had lower k_W_ values in cerebral cortex and white matter, a finding likely preceded by endothelial injury. We found that our cohort, with 76.9% of subjects over 65 years of age reporting at least one vascular risk factor as outlined in Table 1, is generally concordant with national epidemiological data, which reports in US adults over 65 a prevalence of approximately 30% for type 2 Centers for Disease Control and Prevention (2020), 63% for hypertension (Fryar et al., 2017), and 47% for current or former smoking (Kramarow, 2020). Hyperlipidemia, an additional vascular risk factor we assessed in our cohort, has a prevalence of 11% in adults aged 60 and older, and notably, 15% in adults aged 40–59 (Carroll and Hales, 2020).

In our cohort, the mean k_W_ for the cerebral cortex was 81.5 ± 15.5 min^–1^ and white matter was 75.2 ± 13.9 min^–1^, which are lower than the k_W_ values reported by Gold et al. (2021) (98.27 ± 19.8 min^–1^ for whole brain), Fujima et al. (2020) (109.6 ± 28.2 min^–1^), and Shao et al. (2019) (105.0 ± 20.6 min^–1^) (Fujima et al., 2020; Shao et al., 2020; Gold et al., 2021). Differences between these reported values could be attributable to cohort heterogeneity or systemic differences in pulse sequence implementation, acquisition parameters, image reconstruction, and data fitting algorithm. Direct comparison between these DW-ASL techniques in the same subjects is needed to explain this discrepancy.

Many of the findings in our present study showed convergence between k_W_ and CBF values, with notable exceptions. While it is well-documented in larger studies that CBF tends to decrease with age (Buijs et al., 1998; Chen et al., 2011; Zhang et al., 2018), we did not observe a statistically significant correlation. While our relatively small sample size may play a role, our findings also suggest the possibility that k_W_ may be more tightly correlated to age than CBF. We found that CBF in cerebral white matter decreases with WMH burden, replicating other studies (Crane et al., 2015; Stewart et al., 2021). We also reproduced the finding that CBF is decreased within WMH compared to NAWM (Marstrand et al., 2002; Brickman et al., 2009). This convergence of findings between CBF and k_W_ highlights the complex interplay between these two related phenomena. It is possible that decreases in CBF precede changes in k_W_, with chronic hypoxia causing endothelial damage at the BBB. However, the directionality of this relationship cannot be ascertained from this cross-sectional study, and a bidirectional relationship cannot currently be ruled out.

Our pilot study has several limitations. First, our sample size is relatively small and heterogeneous, and may not be reflective of the general population in terms of racial and ethnic diversity. Our findings will need to be confirmed in larger studies incorporating participants from different demographic groups. Second, a small number in our 65+ group indeed may have a degree of undiagnosed mild cognitive impairment, and up to 40% “cognitively normal” elderly adults may have amyloid burden detectable by PET (Mielke et al., 2012), while the amyloid status of our subjects cannot be ascertained, especially since *APOE* genotyping and cerebrospinal fluid sampling was not performed. Nonetheless, no subject had a MoCA score under 20, and no subject had a diagnosed neurodegenerative disorder. Future work will employ more restrictive exclusion criteria to define “cognitively normal,” incorporating more robust neuropsychological testing, amyloid PET, APOE ε4 status, and CSF biomarkers. Third, the brain-blood partition coefficient λ may not be uniform across brain regions. While mapping this tissue parameter is possible (Roberts et al., 1996) and may improve the accuracy of our method, here we follow the recommendation for blood-brain partition coefficient as provided in the most recent consensus paper published by the ISMRM Perfusion Study Group and the European Consortium for ASL in Dementia (Alsop et al., 2015).

While the principal finding that k_W_ is lower in elderly adults needs to be confirmed with longitudinal studies and in a larger cohort, this study represents an important step in understanding the role that the aging BBB plays in neurodegenerative disorders. Our study also calls for the need for more animal and human pathology studies to directly probe the interaction and causal relationships between hypoperfusion, BBB leakage, AQP4 localization, k_W_, glymphatic flow, protein misaggregation, and cognitive dysfunction. With emerging evidence calling into question whether amyloid deposition is the primary driver of AD (Kametani and Hasegawa, 2018; Makin, 2018), studies that aim to illuminate disease pathogenesis will be critical for the development of novel therapies for neurodegenerative disorders.

## Data Availability Statement

The raw data supporting the conclusions of this article will be made available by the authors, without undue reservation.

## Ethics Statement

The studies involving human participants were reviewed and approved by Weill Cornell Medical College IRB. The patients/participants provided their written informed consent to participate in this study.

## Author Contributions

JF, QZ, TN, and JI: conceptualization. QZ and TN: MRI sequence development and QPM scan processing. JF: participant recruitment, enrollment, consent, health questionnaire, and MoCA testing, WMH segmentation, and k_W_ and CBF value extraction in ITK Snap. JF and QZ: QPM scan acquisition. JF and ES statistical analysis. JF and JI: writing—original draft. All Authors: review and editing.

## Conflict of Interest

The authors declare that the research was conducted in the absence of any commercial or financial relationships that could be construed as a potential conflict of interest.

## Publisher’s Note

All claims expressed in this article are solely those of the authors and do not necessarily represent those of their affiliated organizations, or those of the publisher, the editors and the reviewers. Any product that may be evaluated in this article, or claim that may be made by its manufacturer, is not guaranteed or endorsed by the publisher.

## Footnotes

1
www.itksnap.org

## Supplementary Material

Supplementary Figure 1 Univariate linear regression analyses for regional k_W_ and CBF vs. age. There was a statistically significant negative correlation between age and k_W_ in all evaluated regions: cerebral cortex, cerebral white matter (WM), hippocampi, orbitofrontal cortices, and precuneus cortices **(A–E)**. With respect to CBF, there was only a significant negative correlation between cortex and age, and precuneus and age, respectively **(F–J)**.
