# Supplementary figures and images for: Quantitative Water Permeability Mapping of Blood-Brain-Barrier Dysfunction in Aging
Source: Front Aging Neurosci. 2022 Apr 8;14:867452. doi: 10.3389/fnagi.2022.867452 (PMC9024318; doi:10.3389/fnagi.2022.867452)

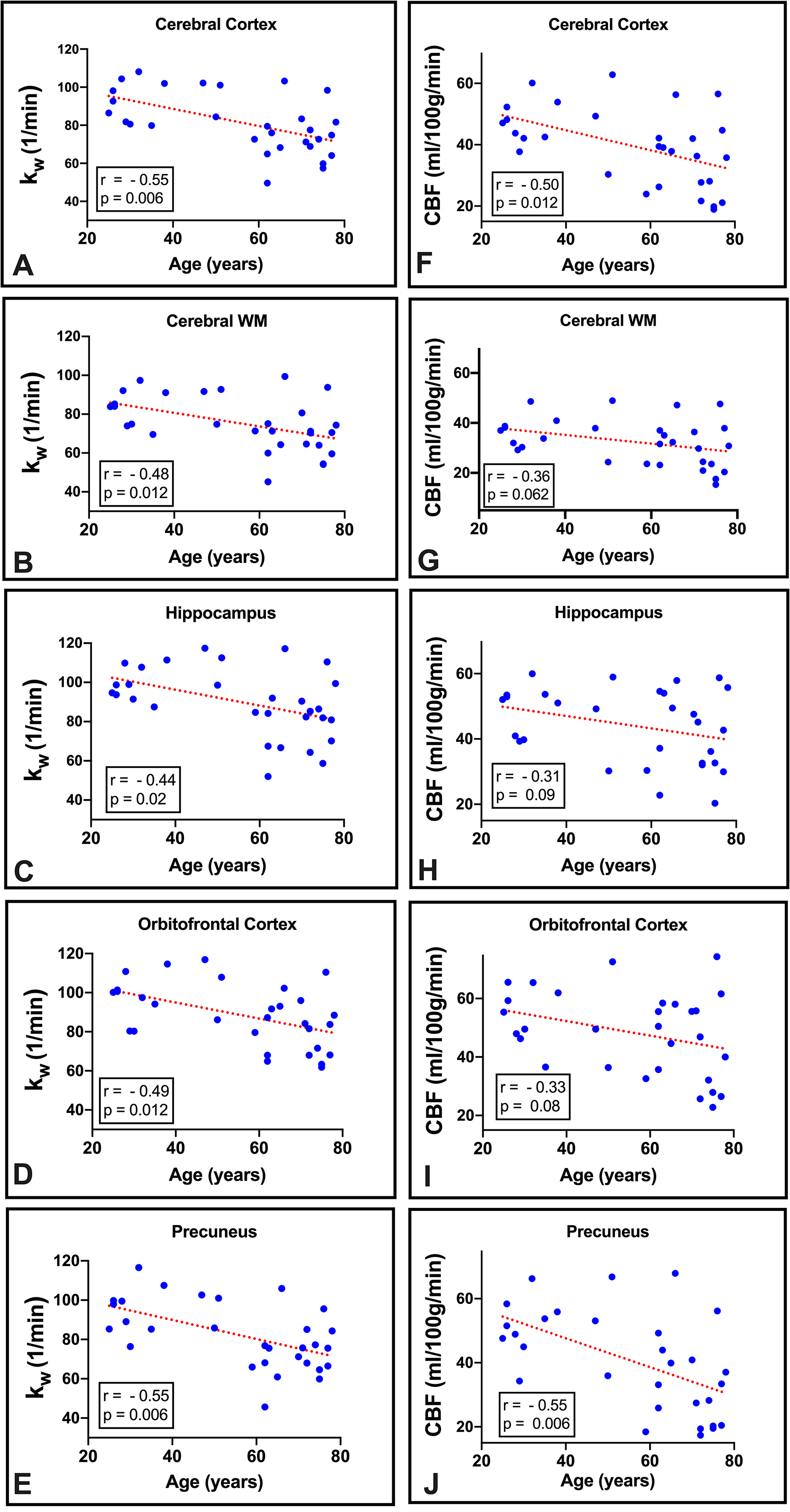

Supplement: Supplementary Figure 1 — Univariate linear regression analyses for regional kW and CBF vs. age. There was a statistically significant negative correlation between age and kW in all evaluated regions: cerebral cortex, cerebral white matter (WM), hippocampi, orbitofrontal cortices, and precuneus cortices (A–E). With respect to CBF, there was only a significant negative correlation between cortex and age, and precuneus and age, respectively (F–J). [file Image_1.tiff]
